# Successful treatment of pulsatile tinnitus caused by jugular bulb diverticula using stent-assisted volumetric coil embolization in a double-jailing technique

**DOI:** 10.1007/s00234-025-03707-w

**Published:** 2025-07-10

**Authors:** Nermin Abozenah, André Kemmling, Simon Klein, Boris Stuck, Mohammad Almohammad

**Affiliations:** 1 Department of Otolaryngology, Head and Neck Surgery, University Hospital of Giessen and Marburg, Philipps-University Marburg, Marburg, Germany; 2 Department of Neuroradiology, University Hospital of Giessen and Marburg, Philipps-University Marburg, Marburg, Germany

**Keywords:** Pulsatile tinnitus, Jugular bulb diverticulum, Stent-assisted embolization, Volumetric coils, Neurointervention

## Abstract

**Background:**

Pulsatile tinnitus (PT) is a distressing auditory symptom, frequently caused by vascular anomalies near the temporal bone. Among the rarer venous causes are jugular bulb diverticula, which can be difficult to diagnose and manage.

**Case presentation:**

We report the case of a patient in their 40s with persistent right-sided pulsatile tinnitus significantly impairing daily functioning. Imaging revealed two diverticula in the right jugular bulb and an enlarged nuchal emissary vein. Conservative treatment was ineffective. Digital subtraction angiography confirmed the venous origin of the tinnitus through provocation testing and temporary balloon occlusion. A novel endovascular approach using volumetric coils and venous double-jailing technique was pursued.

**Intervention:**

Stent-assisted coil embolization was performed using a double-jailing technique. Two PX-SLIM™ microcatheters were jailed through a single ACCERO^®^ Rex stent, enabling precise deployment of Penumbra PC400 volumetric coils into both diverticula and the enlarged nuchal emissary vein. The procedure was completed without complications under general anesthesia.

**Outcome:**

Symptoms resolved immediately and remained absent at follow-up. Dual antiplatelet therapy was tapered to aspirin monotherapy after 12 weeks.

**Conclusion:**

This is the first reported case employing volumetric coils in combination with a venous double-jailing technique for treating jugular bulb diverticula. This approach enabled precise and efficient embolization of multiple venous outpouchings through a single stent construct. The procedure was safe, minimally invasive, and led to complete symptom relief. These findings highlight a promising treatment strategy for selected cases of venous pulsatile tinnitus with complex anatomy.

## Introduction

Pulsatile tinnitus can be defined as the perception of a rhythmic or periodic sound in the absence of any external stimulus [1]. Patients typically perceive pulsatile tinnitus as a heartbeat-like sound in one or both ears. Hearing is usually intact, and the condition is often linked to abnormal blood or cerebrospinal fluid flow near the ear [2]. Vascular causes of pulsatile tinnitus are divided into arterial and venous types. Arterial causes include aberrant or dissected carotid arteries, glomus tumors, fibromuscular dysplasia, and contralateral carotid stenosis leading to increased flow on the affected side [1, 3–5]. Venous causes of pulsatile tinnitus include dural arteriovenous fistulas, venous stenosis, diverticula, a high-riding jugular bulb, and intracranial hypertension [1]. Venous tinnitus can often be distinguished from arterial causes through history and clinical exam, without invasive tests. It tends to be lower in pitch and may improve with neck maneuvers—worsening with contralateral jugular compression and improving with ipsilateral or suboccipital vein compression [4, 6]. Further evaluation of pulsatile tinnitus may require imaging, including high-resolution cranial computed tomography (CT), computed tomography angiography (CTA), magnetic resonance imaging (MRI), magnetic resonance angiography (MRA), carotid Doppler ultrasound, and digital subtraction angiography (DSA) [7]. Sigmoid sinus diverticulum (SSD) is a rare venous cause of pulsatile tinnitus. Its exact cause is unclear, but it may result from sinus wall dehiscence and protrusion into the pneumatised temporal bone [3, 8]. Other rare venous causes of pulsatile tinnitus include jugular bulb anomalies, such as a high-riding bulb, lateral displacement, pathological enlargement, or the presence of a jugular bulb diverticulum [3]. This case illustrates a rare but treatable cause of pulsatile tinnitus—jugular bulb diverticula—and presents a stent-assisted coil embolization using volumetric coils and a venous double-jailing technique. It aims to raise awareness and highlight a minimally invasive therapeutic option.

## Case description

**Fig. 1:**
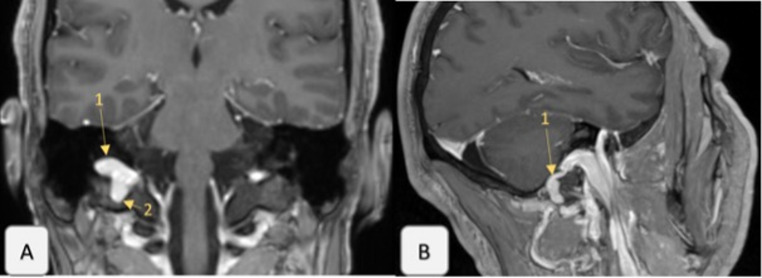
MRI findings of the right jugular bulb before treatment, demonstrating two jugular bulb diverticula and an enlarged nuchal vein outlet. **A**: Coronal T1-weighted MRI after contrast injection, showing the upper diverticulum (1) and the lower diverticulum (2) of the right jugular bulb. Notably, the upper diverticulum extends deeply into the petrous bone toward the cochlea, while the lower diverticulum protrudes deeply into the skull base. **B**: Parasagittal T1-weighted MRI after contrast injection, illustrating the enlarged nuchal vein outlet (1), originating from the right jugular bulb

We report the case of a patient in their 40 s with chronic right-sided pulsatile tinnitus, frontal pressure, and pulsation following influenza with otitis media. Otoscopy and audiometry were normal. Initial treatment for presumed Eustachian tube dysfunction was unsuccessful. A contrast-enhanced MRI in November 2022 excluded arterio-venous malformations but revealed a right-sided jugular bulb diverticulum, identified as the likely source after neuroradiological consultation (Fig. 1).

Due to persistent symptoms and significant distress, digital subtraction angiography (DSA) was performed, focusing on the right-sided vessels near the ear. It revealed two diverticula—cranial and caudal—in the right jugular bulb and an enlarged right nuchal vein outlet. Manipulation (gently rubbing) with a Radifocus™ guidewire reproduced a crackling sound, and contrast injection via a SOFIA™ catheter into the diverticula and the enlarged nuchal elicited the patient’s characteristic low-frequency tinnitus, confirming the venous origin. No similar response was observed in other adjacent areas. emporary balloon occlusion of the jugular bulb fully relieved symptoms, while targeted occlusion of the venous outlet alone resulted in only partial symptom reduction. This supported the involvement of both the diverticula and the emissary vein in the generation of pulsatile tinnitus. Surgical treatment was not considered in this case, as the patient declined an open surgical approach. Following detailed counseling on potential risks and complications—including stroke, vessel dissection, and procedure-related bleeding—stent-assisted coil embolization was subsequently performed with informed consent.

### Description of procedure (Figs. 2 and 3)

**Fig. 2:**
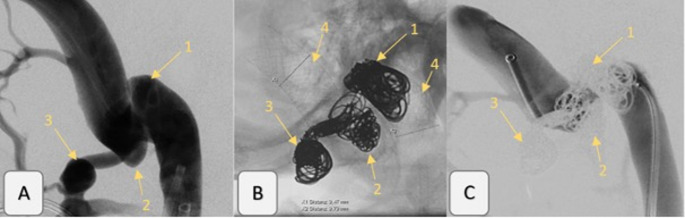
Imaging findings of the right jugular bulb before and after stenting with ACCERO^®^ Rex and coiling of the two jugular diverticula and the enlarged nuchal vein outlet.**A**: Pre-treatment angiogram showing: 1– Upper diverticulum, 2– Lower diverticulum, 3– Enlarged nuchal vein outlet. **B**: Post-treatment unsubtracted image illustrating: 1– Coils in the upper diverticulum, 2– Coils in the lower diverticulum, 3– Coils in the enlarged nuchal vein outlet, 4– ACCERO^®^ Rex stent, extending cranially from the sigmoid sinus to the jugular vein **C**: Post-treatment angiogram demonstrating successful occlusion of the diverticula and the enlarged nuchal vein outlet, with: 1– Coils in the upper diverticulum, 2– Coils in the lower diverticulum, 3– Coils in the enlarged nuchal vein outlet

**Fig. 3:**
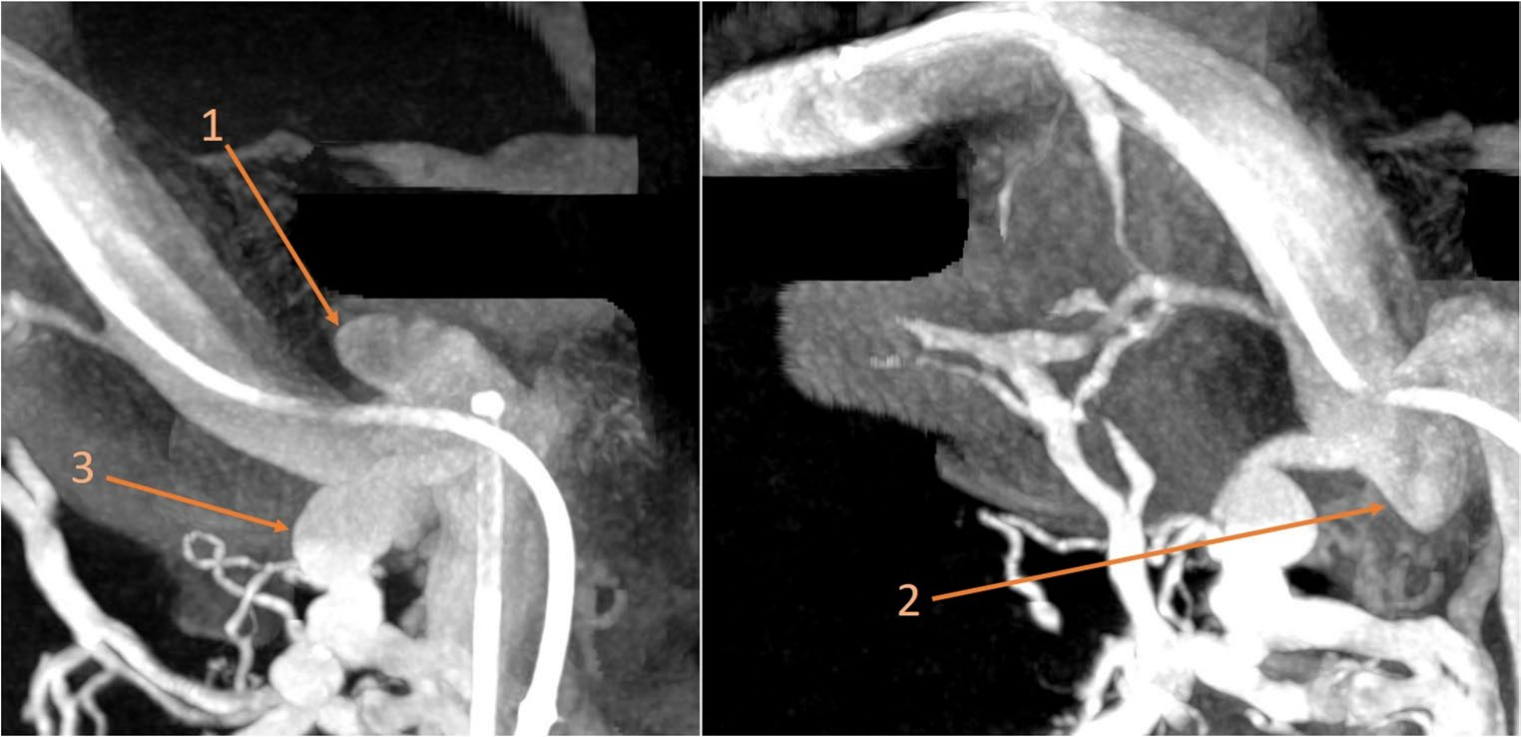
3D rotational angiography findings of the right jugular bulb before treatment, demonstrating two jugular bulb diverticula and an enlarged nuchal vein outlet. 1- The upper diverticulum 2- The lower diverticulum 3- The enlarged nuchal vein outlet

Due to significant anxiety during the initial DSA, the patient declined further procedures under local anesthesia. Given the need for stent-assisted embolization of a wide-necked diverticulum, general anesthesia was chosen to ensure procedural stability and patient comfort. Premedication with prasugrel (10 mg/day) and aspirin (100 mg/day) was initiated five days prior to the intervention. Under general anesthesia and systemic intravenous heparinization with (5000 IU), arterial and venous femoral access were established. A 5 F Radifocus™ catheter (Terumo, Japan) was introduced into the right internal carotid artery, and a Neuron MAX^®^ 088 sheath (Penumbra, USA) was advanced through the femoral vein and placed in the right internal jugular vein. Two PX SLIM™ 0.025” microcatheters (Penumbra, USA) were advanced through the Neuron MAX sheath into the superior and inferior diverticula of the jugular bulb. These were jailed using a 10 × 60 mm ACCERO^®^ Rex stent (Acandis, Germany), which was deployed through a direct puncture of the internal jugular vein via a 5 F short sheath. Volumetric PC400 coils (Penumbra Inc.) were chosen due to their proven benefits, including reduced procedure time, lower radiation exposure, and significantly higher packing density compared to conventional coils, as shown in a study by Berge et al. [9]. These properties were ideal for achieving efficient and durable occlusion in our case. Both diverticula were successfully occluded, utilizing a double-jailing technique. Additionally, the enlarged nuchal vein outflow was coiled using the same coils. All catheters were removed under continuous aspiration without complications.

The patient experienced immediate symptom relief, confirming the venous origin. Dual antiplatelet therapy was tapered to aspirin monotherapy after 12 weeks. At follow-up, tinnitus had fully resolved, confirming procedural success.

## Discussion

Pulsatile tinnitus (PT) is an uncommon auditory phenomenon, often stemming from vascular anomalies that generate turbulent or altered blood flow near the temporal bone. While arterial etiologies such as arteriovenous fistulas, glomus tumors, or carotid dissections are more commonly reported, rare venous sources—including sigmoid sinus diverticula and jugular bulb anomalies—are increasingly recognized as treatable causes of PT [10].

Surgical treatment, such as transmastoid resurfacing of the jugular bulb, has been reported as an effective option in selected cases of pulsatile tinnitus, particularly when endovascular access is limited or bony dehiscence is present [11].

Endovascular therapy has become a preferred treatment option for pulsatile tinnitus of vascular origin, owing to its technical simplicity and low complication rate. Multiple case reports have shown that coil embolization and stent-assisted techniques are effective, particularly for venous anomalies such as sigmoid sinus and jugular bulb diverticula, often resulting in complete or significant symptom relief [1, 2, 6, 7, 12–15].

A case report even described successful treatment of pulsatile tinnitus caused by a jugular bulb diverticulum using an unconventional device—the Woven EndoBridge (WEB) device [3].

In our case, chronic right-sided pulsatile tinnitus due to two jugular bulb diverticula and an enlarged nuchal emissary vein was successfully treated with stent-assisted coil embolization. To our knowledge, this is the first reported case utilizing volumetric coils, which help reduce both procedural time and radiation exposure [9]. Additionally, it is the first to apply the venous double-jailing technique with two 0.025” microcatheters jailed simultaneously through a single stent, allowing targeted coil deployment in both diverticula. This approach may offer specific advantages in patients with complex venous anatomy. As the patient explicitly declined surgical management, favoring a minimally invasive solution, this approach provided an effective and well-tolerated alternative.

### Limitations

This case report is limited by the absence of objective tinnitus assessment, the lack of long-term follow-up, and its inherently anecdotal nature. The applied technique was tailored to an individual anatomical setting and patient preference, which strongly limits its generalizability. Further clinical experience is needed to validate its broader applicability.

### Conclusion

This case demonstrates that pulsatile tinnitus caused by jugular bulb diverticula and an enlarged emissary vein could be effectively treated using a minimally invasive endovascular approach. Building on previous reports of promising outcomes with endovascular techniques for venous pulsatile tinnitus, this case illustrates the successful use of a double-jailing stent-assisted strategy combined with high-volume volumetric coils. The approach enabled precise and efficient occlusion of multiple venous outpouchings through a single stent construct and led to complete symptom resolution. While these results are encouraging, the technique should be considered on a case-by-case basis, particularly in patients with complex venous anatomy. Further experience and long-term data are needed to better define its role and to guide material selection in similar clinical scenarios.

## Data Availability

No datasets were generated or analysed during the current study.

## Abbreviations

CT: Computed Tomography
CTA: Computed Tomography Angiography
DSA: Digital Subtraction Angiography
ICJ: Internal Jugular Vein
ICA: Internal Carotid Artery
IU: International Units
MRA: Magnetic Resonance Angiography
MRI: Magnetic Resonance Imaging
PC400: Penumbra Coil 400
PX SLIM™: Delivery Microcatheter
PT: Pulsatile tinnitus
SSD: Sigmoid Sinus Diverticulum
